# Phospho-ibuprofen (MDC-917) suppresses breast cancer growth: an effect controlled by the thioredoxin system

**DOI:** 10.1186/bcr3105

**Published:** 2012-01-31

**Authors:** Yu Sun, Leahana M Rowehl, Liqun Huang, Gerardo G Mackenzie, Kvetoslava Vrankova, Despina Komninou, Basil Rigas

**Affiliations:** 1 Division of Cancer Prevention, Department of Medicine, Stony Brook University, Stony Brook, NY 11794-8173, USA; 2 Medicon Pharmaceuticals, Inc., 25 Health Sciences Drive, Stony Brook, NY 11790-3350, USA

## Abstract

**Introduction:**

We have recently synthesized phospho-ibuprofen (P-I; MDC-917), a safer derivative of ibuprofen, which has shown anti-cancer activity. We investigated its efficacy and mechanism of action in the treatment of breast cancer in preclinical models.

**Methods:**

We evaluated the anti-breast-cancer efficacy of P-I alone or incorporated into liposomes (Lipo-P-I) in human estrogen receptor-positive (MCF-7) and triple-negative, i.e., estrogen receptor-negative, progesterone receptor-negative and HER2-negative (MDA-MB231) breast cancer cell lines - as they represent the most frequent (estrogen receptor-positive) and the most difficult-to-treat (triple-negative) subtypes of breast cancer - and their xenografts in nude mice. We assessed the effect of P-I on the levels of reactive oxygen nitrogen species in response to P-I using molecular probes, on the thioredoxin system (expression and redox status of thioredoxin-1 (Trx-1) and thioredoxin reductase activity), on cyclooxygenase 2, NF-κB and mitogen-activated protein kinase cell signaling; and on the growth of xenografts with stably knocked-down Trx-1.

**Results:**

Compared with controls, P-I 400 mg/kg/day inhibited the growth of MDA-MB231 xenografts by 266%, while the growth of MCF-7 xenografts was inhibited 51% byP-I 300 mg/kg/day and 181% by Lipo-P-I 300 mg/kg/day. In both cell lines, P-I induced oxidative stress and suppressed the thioredoxin system (oxidized Trx-1 and decreased its expression; inhibited thioredoxin reductase activity). These changes triggered downstream redox signaling: the activity of NF-κB was suppressed and the Trx-1-ASK1 complex was dissociated, activating the p38 and JNK mitogen-activated protein kinase cascades. Trx-1 knockdown abrogated the anti-cancer effect of P-I *in vitro *and *in vivo*.

**Conclusion:**

P-I is safe and effective against breast cancer. Liposomal formulation enhances its efficacy; the effect is heavily dependent on the induction of oxidative stress and the suppression of the thioredoxin system. P-I merits further evaluation as an agent for the treatment of breast cancer.

## Introduction

Breast cancer is the most frequently diagnosed cancer and the leading cause of cancer death among females owing, to a large extent, to the lack of effective and safe agents [1]. Phospho-ibuprofen (P-I; MDC-917) is a novel derivative of ibuprofen with significant efficacy against colon cancer and a favorable safety profile [2,3]. Our preliminary data indicated that P-I might be effective in the treatment of breast cancer. Given the need for new agents for the control of breast cancer, we undertook a systematic study of the effect of P-I in breast cancer.

Thioredoxin (Trx), thioredoxin reductase (TrxR), and nicotinamide adenine dinucleotide phosphate comprise the Trx system, which is crucial to redox homeostasis [4-7]. The thioredoxin-1 (Trx-1) isoform of Trx, the main intracellular antioxidant oxidoreductase [8-10], is normally in its reduced state (Trx-1-(SH)_2_), defined primarily by two vicinal cysteine thiol groups at its active site (Cys32 and Cys35). When one of its client cellular proteins is oxidized, Trx-1-(SH)_2 _reduces them, while itself paying the price of becoming oxidized in the process to Trx-1-S_2_. Normally, Trx-1-S_2 _is rapidly restored to its functional reduced status (Trx-1-(SH)_2_) by TrxR and nicotinamide adenine dinucleotide phosphate.

The role of Trx-1 in breast cancer is not completely understood. Oxidative stress and activation of redox signaling pathways accompany breast cancer carcinogenesis and are correlated with prognosis in breast cancer patients [11]. As a rapid response molecule to oxidative stress, Trx-1 modulates redox signaling pathways via thiol-disulfide exchange with redox-responsive molecules, such as the transcription factors Ref-1 and NF-κB [9,12,13], MAP3K5/apoptosis signal-regulating kinase 1 (ASK1) [14], and the Trx-1 interacting protein (TXNIP) [10,15]. The end result of these effects is modulation of cell kinetics, which can sometimes, as we demonstrate here, culminate in inhibition of cell growth and/or induction of apoptosis. Another recently appreciated consequence of oxidative stress is the induction of endoplasmic reticulum stress, which links it to inflammation, with significant implications for several disorders including cancer [16,17].

The level of Trx-1 is overexpressed in human breast carcinoma compared with normal breast tissue and has been associated with breast cancer progression [18]. Furthermore, overexpression of Trx-1 or TrxR has been related to resistance to chemotherapy [19]. All of these findings underscore the crucial role of the Trx system in breast cancer and establish it as a target for drug development [5,20,21]. In this article, we report the strong efficacy of P-I against breast cancer and establish the critical role of the Trx system in mediating its anti-cancer effect through changes in downstream redox-responsive signaling pathways.

## Materials and methods

### Liposome-encapsulated phospho-ibuprofen

Liposome-encapsulated phospho-ibuprofen (Lipo-P-I) was generated following standard procedures by Encapsula NanoSciences LLC (Nashville, TN, USA). The formulation is L-α-phosphatidylcholine (80 mg/ml), PEG-2000-DSPE (14.8 mg/ml) and P-I (45 mg/ml). The particle size is 200 nm. The concentration of liposomal P-I was determined by HPLC before use [2].

### Cell culture and cell viability and cytokinetic assays

We used MCF-7 and MDA-MB231 human breast carcinoma cell lines, which reflect, to a large extent, the major features of cancer cells *in vivo *[22]. Estrogen receptor (ER)-positive MCF-7 cells are human breast epithelial adenocarcinoma cells derived from the metastatic pleural effusion of a breast adenocarcinoma patient. This cell line retains several characteristics of differentiated mammary epithelium, including the ability to process estradiol via cytoplasmic ERs and the capability of forming domes [23]. Triple-negative (ER-negative, progesterone receptor-negative and HER2-negative) MDA-MB-231 cells were obtained from a pleural effusion of a patient who had developed a 'poorly-differentiated tumor tending toward papillary configuration and tubule formation', while also having an intraductal carcinoma [24].

These cell lines were grown as recommended by the American Type Culture Collection (Manassas, VA, USA), seeded at 5 × 10^4 ^cells/cm^2 ^for 24 hours and then treated as indicated. As previously described [25], we determined the cell viability by the MTT assay (Roche Diagnostics, Indianapolis, IN, USA), apoptosis by flow cytometry following staining with annexin V-FITC/propidium iodide, the cell cycle after staining with propidium iodide, and cell proliferation by the bromodeoxyuridine method.

### Determination of reactive oxygen nitrogen species

We assayed the following, as previously described [25]: reactive oxygen nitrogen species (RONS), using the general probe 2',7'-dichlorofluorescein diacetate (DCFDA); superoxide anion (O_2_^-^) in whole cells, by staining with 5 μM dihydroethidium and analyzing fluorescence using flow cytometry; and mitochondrial O_2_^-^, by seeding cells in glass bottom culture dishes, staining with MitoSOX™ Red (Life Technologies, Grand Island, NY, US) and examining them under a Zeiss LSM510 Meta NLO confocal microscope (Carl Zeiss Microscopy, LLC, Thornwood, NY, USA).

### Thioredoxin redox status assay

After each treatment, 10^6^cells were lysed in 6 M guanidinium chloride, 50 mM Tris/HCl, pH 8.3, 3 mM ethylenedinitrilotetraacetic acid, 0.5% Triton X-100 containing 50 mM iodoacetic acid [26]. After 30 minutes at 37°C, excess iodoacetic acid was removed using Microspin G-25 columns (GE Healthcare Life Sciences, Pittsburgh, PA, USA). Oxidized and reduced Trx-1 were separated by native PAGE, electroblotted onto a nitrocellulose membrane and probed with anti-Trx-1 antibody.

### Thioredoxin reductase activity

TrxR activity was determined in the protein lysate using a commercially available kit, as per the instructions of the manufacturer (Cayman Chemical Company, Ann Arbor, MI, USA). In this assay, TrxR uses nicotinamide adenine dinucleotide phosphate to reduce 5,5-dithiobis-2-nitrobenzoic acid to 5-thio-2-nitrobenzoic acid.

### Immunoblotting and electrophoretic mobility shift assay

After each treatment, cell proteins were fractionated by SDS gel electrophoresis and immunoblotted with antibodies against Trx-1 (AbCam, Cambridge, MA, USA), B-cell lymphoma 2 (Bcl-2) and myeloid cell leukemia 1 (Mcl-1) (Santa Cruz Biotechnology, Santa Cruz, CA, USA), and ASK1, phospho-p38, phospho-extracellular signal-regulated kinase (phospho-ERK) and phospho-Jun N-terminal kinase (phospho-JNK) (Cell Signaling, Danvers, MA, USA) following standard procedures. For the NF-κB electrophoretic mobility shift assay, nuclear extracts obtained as described elsewhere [27] were analyzed using the Gel Shift Assay System (Promega Corporation, Madison, WI, USA), as described previously [25].

### Assay for apoptosis signal-regulating kinase 1-thioredoxin-1 complex formation

The ASK1-Trx-1 complex was immunoprecipitated using Protein A/G PLUS-Agarose beads following the manufacturer's instructions (Santa Cruz Biotechnology) as described [25].

### siRNA silencing of the thioredoxin gene and generation of stable Trx-1 knockdown MCF-7 cells

MCF-7 or MDA-MB231 cells (0.8 × 10^5^) were transfected with 100 nM Trx siRNA or control siRNA (Dharmacon Inc., Chicago, IL, USA) for 72 hours using lipofectamine 2000 (Life Technologies, Grand Island, NY, USA). For Trx-1 stable knockdown, we used one control particle and three Trx-1 shRNA SMARTvector 2.0 Lentiviral particles (Thermo Scientific Dharmacon Inc., Chicago, USA). Then 1.5 × 10^6 ^MCF-7 cells at passage six were incubated overnight with a 4.5 × 10^5 ^titer of lentivirus particles (0.3 multiplicity of infection/cell) suspended in growth medium with 3 μg/ml polybrene. The infected cells were passaged three times in selection medium containing 1 μg/ml puromycin, and were assayed for transfection efficiency by immunoblot for Trx-1 and by flow cytometry for endogenous GFP expression. Aliquoted positive cells were stored at -80°C. Three Trx-1 knockdown cell lines (C1, C2, and C3) were generated; in all *in vivo *experiments we used cell line C3 and control cells, designated MCF-7^shTrx-1 ^and MCF-7^shControl^, respectively.

### *In vivo *studies

All animal experiments were approved by the Institutional Animal Care and Use Committee. Breast cancer cells (1.5 × 10^6^) were xenografted subcutaneously in both flanks of 5-week-old to 6-week-old female Balb/C nude mice as described previously [2,28]. Three days before implanting MCF-7 cells, mice received a 0.72 mg β-estradiol pellet (Innovative Research of America, Sarasota, FL, USA) subcutaneously in their front-back area. Vehicle, P-I or Lipo-P-I administration (once a day, 5 days/week) started when the average tumor volume of MCF-7 xenografts was 160 mm^3 ^and that of MDA-MB231 xenografts was 100 mm^3^. Xenograft growth inhibition in response to treatment was calculated by comparing between drug-treated and vehicle-treated group the difference in percentage increase of tumor volume from the zero-time value to that at sacrifices.

Cell death and proliferation in xenografts were determined by TUNEL and Ki-67 immunostaining, as described [29]. Cyclooxygenase-2 (COX-2) expression, NF-κB activation and Trx-1 expression were determined by immunohistochemical staining following standard protocols (antibodies from Cell Signaling and Abcam, respectively) as reported previously [29]. For scoring from each slide, we photographed three to five randomly selected fields (200×). For each of these parameters (TUNEL, Ki-67, COX-2, NF-κB and Trx-1), the number of positive cancer cells and the number of all (positive and negative) cancer cells were determined for each photograph by two pathologists blinded to their identity and the percentage of positive cells was calculated based on the average of the values generated by the two pathologists (maximal variation 4.2%).

### Quantitative PCR of Trx-1 mRNA level from Trx-1 knockdown and control MCF-7 xenografts

Total RNA was isolated using TRIZOL reagent (Invitrogen) from xenografts. The quantitative PCR of Trx-1 mRNA was determined using SYBR Green PCR master mix (A&B Applied Biosystems, Carlsbad, CA, USA) in accordance with the manufacturer's protocol [30].

### Statistical analyses

Results are expressed as the mean ± standard error of the mean. Differences between groups were determined by the Student's *t *test. *P *< 0.05 was statistically significant.

## Results

### Phospho-ibuprofen inhibits the growth of human breast cancer cells and xenografts

We initially determined the growth inhibitory effect of P-I on human breast cancer cell lines representing its major clinical subtypes. The 24-hour values for the concentration that inhibits cell growth by 50% (IC_50_) for P-I were as follows: ER-positive: MCF-7 = 79 *± *5.6 μM; HER2-positive: AU-565 = 198 *± *8.4 μM; and triple-negative: BT-549 = 127 *± 2*.3 μM, MDA-MB231 = 28 *± *2.7 μM, and BT-20 = 89 ± 3.5 μM.

**Figure 1:**
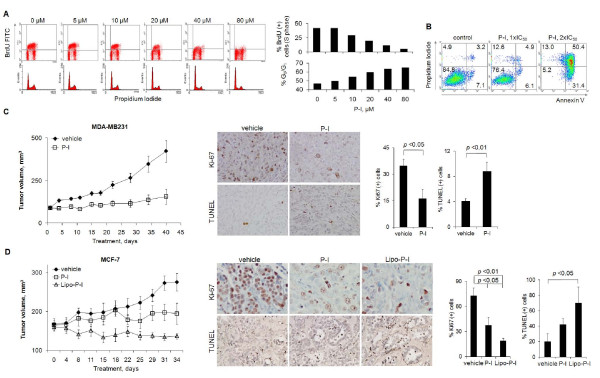
**Phospho-ibuprofen inhibited the growth of breast cancer cells and xenografts**. **(A) **Proliferation (BrdU/propidium iodide staining; upper panel) and cell cycle (propidium iodide staining; lower panel) of MCF-7 cells treated with phospho-ibuprofen (P-I) was evaluated by flow cytometry and the results were quantified (right panel). **(B) **Apoptosis of MCF-7 cells treated with P-I was evaluated by annexin V and propidium iodide staining. Tumor growth curves of **(C) **MDA-MB231 xenografts (treated with P-I 400 mg/kg/day or vehicle) and **(D) **MCF-7 xenografts (treated with P-I 300 mg/kg/day, liposome-encapsulated phospho-ibuprofen (Lipo-P-I) 300 mg/kg/day, or vehicle) are shown. Quantification of cell proliferation (Ki67) or cell death (TUNEL) staining is shown on the right of their representative images (200× magnification) as indicated. IC_50_, concentration that inhibits cell growth by 50%.

We also determined the cytokinetic effect of P-I on breast cancer cells and its ability to inhibit the growth of breast cancer xenografts. P-I decreased the proliferation, blocked the G_1_→S cell cycle transition and induced apoptosis in MCF-7 cells (Figure 1A, B). Similar results were obtained with MDA-MB231 cells (Additional file 1). Mice with MDA-MB231 xenografts were treated for 40 days with vehicle or P-I 400 mg/kg/day, starting when the average tumor volume was ~100 mm^3^. At the end of the study, the tumor volume of controls was 423 ± 64 mm^3 ^and that of P-I-treated mice was 157 ± 44 mm^3 ^(*P *< 0.01; Figure 1C). P-I 400 mg/kg/day therefore inhibited the growth of MDA-MB231 xenografts by 266%.

The method of delivery can affect the pharmacological effect of a given drug [31]. For example, doxorubicin in liposomes is more efficacious than free doxorubicin [32,33]. Such enhanced efficacy is explained by the enhanced permeability and retention effect, which represents the preferential uptake of liposomes by tumor cells due to the leakiness of the vessels traversing a tumor [34,35]. We therefore studied the anti-breast cancer effect of P-I formulated in liposomes.

Mice with MCF-7 xenografts were treated for 34 days with P-I 300 mg/kg/day or Lipo-P-I 300 mg/kg/day or with vehicle, starting when the average tumor volume was ~160 mm^3^. Compared with controls, P-I inhibited tumor growth significantly, starting on day 29 and continuing until sacrifice when the tumor volume was 276 ± 23 mm^3 ^and 195 ± 27 mm^3^, respectively (*P *< 0.05; Figure 1D). The inhibitory effect of Lipo-P-I became statistically significant starting on day 8 of treatment. At sacrifice, xenografts had regressed compared with baseline, being 14% smaller (137 ± 7 mm^3 ^vs. 160 ± 9 mm^3^; *P *= 0.09, significant for trend). Lipo-P-I was more efficacious than P-I (*P *< 0.05).

We also determined cell proliferation and cell death in xenografts using Ki-67 staining and the TUNEL assay, respectively (Figure 1C, D). In MDA-MB231 and MCF-7 xenografts, P-I inhibited proliferation (*P *< 0.05 and *P *< 0.05) and increased apoptosis (*P *< 0.01 and *P *= 0.08, significant for trend). Both effects were more pronounced in Lipo-P-I-treated animals (*P *< 0.01 and *P *< 0.05, respectively, compared with control).

### Effect of phospho-ibuprofen on reactive oxygen nitrogen species and glutathione levels in MCF-7 and MDA-MB231 cells

**Figure 2:**
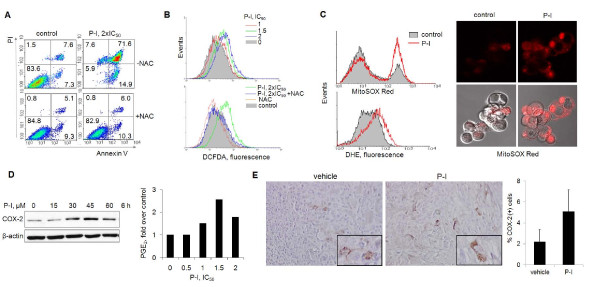
**Effect of phospho-ibuprofen on reactive oxygen and nitrogen species and cyclooxygenase-2 levels in breast cancer**. **(A) **Pretreating MCF-7 cells with 15 mM *N*-acetyl-cysteine (NAC) suppressed phospho-ibuprofen (P-I)-induced apoptosis. **(B) **After 1 hour of treatment, MCF-7 cells as indicated were stained with 2',7'-dichlorofluorescein diacetate (DCFDA) and the fluorescent intensity was determined by flow cytometry. IC_50_, concentration that inhibits cell growth by 50%. **(C) **Left: After 1 hour of treatment with P-I 1.5×IC_50_, MCF-7 cells were stained with MitoSOX Red or dihydroethidium (DHE) and the fluorescent intensity was determined by flow cytometry. Right: Confocal microscopy of MitoSOX Red-stained MCF-7 cells after same treatment as above. Upper panel: MitoSOX Red alone. Lower panel: MitoSOX Red merged with DIC images. **(D) **Cyclooxygenase-2 (COX-2) levels in MDA-MB231 cells were detected by immunoblot; loading control, β-actin. After a 6-hour treatment with P-I, prostaglandin E_2 _(PGE_2_) levels were assayed in the culture medium of MDA-MB231 cells using a kit from Cayman Chemicals. Results are folds over control. (**E**) Representative images (200× magnification) from immunohistochemistry-stained COX-2 in MDA-MB231 xenografts (from Figure 1C) and their quantification are shown. Insets: 600× images of COX-2-positive cells.

RONS are important early mediators of the anti-cancer effect of modified nonsteroidal anti-inflammatory drugs (NSAIDs) [36]. Pretreatment of MCF-7 cells with the antioxidant *N*-acetyl-cystein (NAC) blocked P-I-induced apoptosis by 70.2% (Figure 2A), indicating that this effect is redox dependent. We thus evaluated whether P-I induces RONS in breast cancer cells using the molecular probes DCFDA (detects > 10 individual RONS [37,38]), dihydroethidium (detects O_2_^-^) or MitoSOX Red (specific for mitochondrial O_2_^-^).

**Figure 3:**
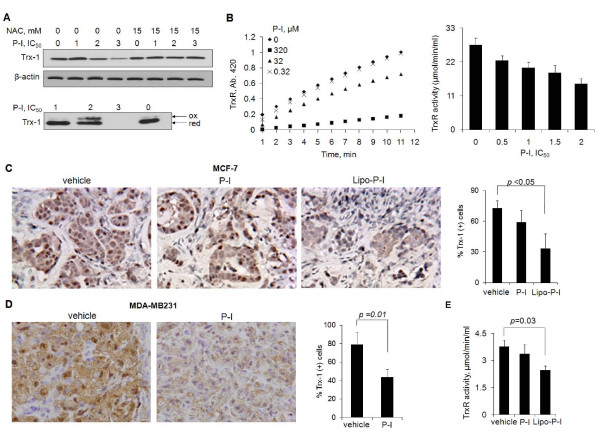
**Effect of phospho-ibuprofen on the thioredoxin system in breast cancer**. **(A) **Upper panel: Thioredoxin-1 (Trx-1) levels in MCF-7 cell lysates were determined by immunoblot. Lower panel: The redox status of Trx-1 in MCF-7 cells was assayed as in Materials and methods after 1 hour of treatment with phospho-ibuprofen (P-I). IC_50_, concentration that inhibits cell growth by 50%; NAC, *N*-acetyl-cysteine; ox, Trx-1 oxidized form; red, Trx-1 reduced form. **(B) **P-I reduces thioredoxin reductase (TrxR) activity. The effect of P-I on the enzymatic activity of TrxR was assayed *in vitro *using TrxR purified from rat liver (left). The TrxR activity was determined in protein extracts from MCF-7 cells treated with P-I for 1 hour (right). Representative images (200× magnification) of Trx-1 in **(C) **MCF-7 or **(D) **MDA-MB231 xenografts by immunohistochemistry staining and quantifications are shown. Lipo-P-I, liposome-encapsulated phospho-ibuprofen. **(E) **TrxR activity was determined in MCF-7 tumor lysates.

In cell lines, P-I concentration-dependently induced RONS detected by DCFDA, an effect abrogated by pretreating the cells with 15 mM NAC (Figure 3B). O_2_^- ^levels were significantly increased in the entire cell (dihydroethidium probe) and even more so in mitochondria (MitoSox Red; Figure 3C). P-I decreased modestly the levels of glutathione, the major intracellular antioxidant [39], and only at concentrations exceeding its IC_50 _for cell growth (Additional file 2).

### Effect of phospho-ibuprofen on the eicosanoid pathway

COX-2 is a well-recognized biochemical target of NSAIDs, explaining part of their anti-inflammatory effects. P-I inhibits the production of prostaglandin E_2 _in NIH3T3 cells, a mouse embryonic fibroblast cell line [2]. Furthermore, COX-2 inhibition by NSAIDs is considered one of the mechanisms by which these drugs prevent cancer [40], although COX-independent effects are well described [41,42]. We have shown that COX-2 induction results from oxidative stress in cancer cells when treated with NSAID derivatives [41]. However, P-I affected COX-2 expression *in vitro *in a cell-type-dependent manner. It suppressed COX-2 expression in MCF-7 cells, while stimulating COX-2 expression and prostaglandin E_2 _production in MDA-MB231 cells (Figure 2D, Additional file 2). The change of COX-2 level was thus independent of RONS in P-I-treated breast cancer cells. More importantly, P-I did not significantly change COX-2 expression in xenografts (*P *> 0.05; Figure 2E). We therefore conclude that the anti-cancer effect of P-I is COX-2 independent.

### Phospho-ibuprofen modulates the thioredoxin system in breast cancer

The Trx system plays a crucial role in maintaining the redox homeostasis of cells by reducing oxidized proteins; such protein oxidation can occur during oxidative stress [43]. In view of the increased RONS levels in response to P-I, we determined the status of Trx-1, the main isoform of Trx, and of TrxR in cultured breast cancer cells and in their xenografts in mice.

In MCF-7 cells after 1 hour of treatment, P-I decreased the levels of Trx-1 - very modestly at concentrations corresponding to 2×IC_50 _and significantly at those corresponding to 3×IC_50_. Pretreatment with 15 mM NAC restored the level of Trx-1, indicating that RONS induction is upstream of Trx-1 (Figure 3A). P-I at 2×IC_50 _also promoted the oxidation of Trx-1, with about one-third of the protein being oxidized. Similar results were obtained in MDA-MB231 cells (Additional file 3). P-I failed to affect TrxR expression but inhibited its enzymatic activity. In an *in vitro *assay - which uses purified TrxR from rat liver to assess its ability to reduce the oxidized substrate 5,5'-dithiobis-2-nitrobenzoic acid - P-I suppressed the activity of TrxR (IC_50 _= 88 μM). Similarly, the activity of TrxR determined in protein extracts from MCF-7 cells was suppressed following treatment with P-I (Figure 3B).

We examined the expression of Trx-1 in MCF-7 and MDA-MB231 xenografts from the efficacy study mentioned above. Immunohistochemistry staining showed cytosolic and nuclear expression of Trx-1 in both types of tumors (Figure 3C, D). Compared with controls, P-I in MDA-MB231 tumors decreased Trx-1 expression by 47.4% (41.5 ± 8.2 vs. 78.9 ± 12.9, *P *= 0.01). In MCF-7 tumors, free P-I had no significant effect on Trx-1 expression, but Lipo-P-I decreased it by 54.1% (33.3 ± 14 vs. 72.6 ± 7.4, *P *< 0.05). Of note, the dose of P-I in animals with MCF-7 xenografts was 300 mg/kg/day and in those with MDA-MB231 tumors was 400 mg/kg/day. This may account for the difference in the inhibition of Trx-1 expression. Although free P-I did not inhibit TrxR activity in either type of xenograft, Lipo-P-I reduced the activity significantly by 35% in MCF-7 xenografts (*P *= 0.03; Figure 3C, D).

### Phospho-ibuprofen modulates thioredoxin-dependent cell signaling

NF-κB and mitogen-activated protein kinases (MAPKs) are major determinants of cell renewal and cell death [24,25]. The Trx system is intimately linked to these two signaling pathways. Trx-1 enhances the binding of NF-κB to DNA by reducing the intermolecular Cys62 -S-S- bond of its p50 subunit [12]. Trx-1 controls the activation of JNK and p38 by binding to ASK1 [26]. When Trx-1 is oxidized, ASK1 dissociates from it, autophosphorylates, and activates MAPK cascades. We thus examined whether the effect of P-I on the Trx system modulates these redox signaling pathways.

**Figure 4:**
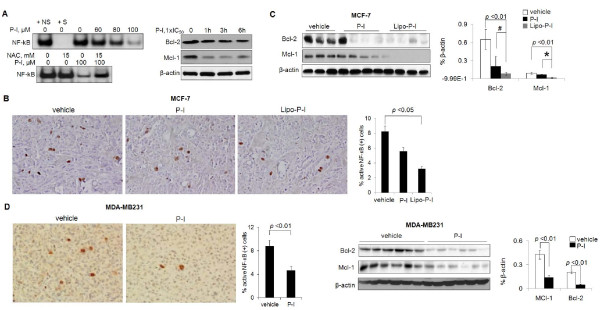
**Phospho-ibuprofen inhibited NF-κB signaling in breast cancers**. **(A) **Left panels: NF-κB-DNA binding was determined by electrophoretic mobility shift assay (EMSA) in nuclear fractions of MCF-7 cells treated with or without phospho-ibuprofen (P-I). 0, control nuclear fraction incubated with 100-fold molar excess of specific (+S) or nonspecific (+NS) unlabeled oligonucleotide containing the consensus sequence of NF-κB. IC_50_, concentration that inhibits cell growth by 50%; NAC, *N*-acetyl-cysteine. Right panels: Immunoblots of B-cell lymphoma 2 (Bcl-2) and myeloid cell leukemia 1 (Mcl-1) from MCF-7 cells treated with or without P-I as indicated. Representative images (200×) of immunohistochemistry staining of activated NF-κB in **(B) **MCF-7 and **(D) **MDA-MB-231 xenografts (as in Figure 1) and quantification results are shown. Immunoblots of Bcl-2 and Mcl-1 from the lysates of **(C) **MCF-7 and **(D) **MDA-MB231 xenografts (as indicated) are shown. Lipo-P-I, liposome-encapsulated phospho-ibuprofen. #*P *< 0.05; *P < 0.001. Loading control, β-actin.

P-I inhibited NF-κB activation in MCF-7 cells, whereas 1-hour pretreatment with 15 mM NAC restored the NF-κB activity to control level (Figure 4A). A similar inhibition was observed in MCF-7 xenografts (Figure 4B). P-I inhibited NF-κB activation by 32% (*P *= 0.08 significantly for trend) and Lipo-P-I by 61% (*P *< 0.05). Both *in vitro *and *in vivo*, P-I suppressed the expression of Bcl-2 and Mcl-1, two anti-apoptotic proteins transcriptionally regulated by NF-κB [44,45] (Figure 4C). Similar results were found in MDA-MB231 xenografts (Figure 4D).

**Figure 5:**
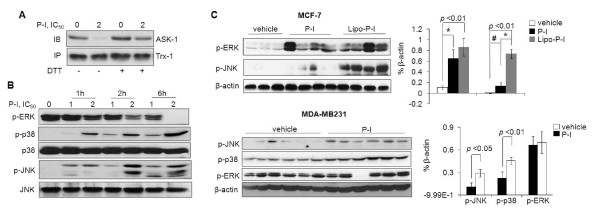
**Phospho-ibuprofen-modulated mitogen-activated protein kinases in breast cancers**. **(A) **Phospho-ibuprofen (P-I) dissociates apoptosis signal-regulating kinase 1 (ASK1)-thioredoxin-1 (Trx-1) complex in MCF-7 cells. Total cell lysates from vehicle-treated or P-I-treated cells were immunoprecipitated using anti-Trx-1 antibody in the presence (+) or absence (-) of dithiothreitol (DTT). Supernatants (IB) and immunoprecipitants (IP) were immunoblotted with anti-ASK1 and anti-Trx-1, respectively. IC_50_, concentration that inhibits cell growth by 50%. **(B) **Mitogen-activated protein kinase (MAPK) activity was determined by immunoblotting MCF-7 cell lysates with antibodies against phosphorylated or total MAPKs as indicated. **(C) **Effect of P-I on MAPK signaling in breast cancer xenografts. Protein lysates from MCF-7 xenografts (upper) or from MDA-MB231 xenografts (lower) as described in Figure 1 were immunoblotted with antibodies against phosphorylated MAPKs. Loading control, β-actin. Lipo-P-I, liposome-encapsulated phospho-ibuprofen. Results were quantified and graphed on the right. #*P *< 0.05; *P < 0.001.

P-I dissociated the ASK1-Trx-1 complex nearly completely in MCF-7 cells (Figure 5A). Incubation of the cell lysate with the reducing agent dithiothreitol restored the complex, indicating that the dissociation of ASK1 from Trx-1 was due to P-I-induced oxidation of Trx-1. We therefore studied the downstream ASK1-MKK4-p38MAPK/JNK signaling. In both cell lines, P-I activated p38 and JNK (increasing their phosphorylation) time and concentration dependently. However, P-I suppressed ERK activation, particularly at a high concentration (2×IC_50_). The total levels of these MAPKs were not changed by P-I (Figure 5B). In MCF-7 xenografts, P-I activated JNK and ERK but not p38. In MDA-MB231 xenografts, P-I activated JNK and p38 but not ERK (Figure 5C). Such differential activation of MAPKs underscores their context dependence.

### Thioredoxin-1 mediates the anti-cancer effect of phospho-ibuprofen

**Figure 6:**
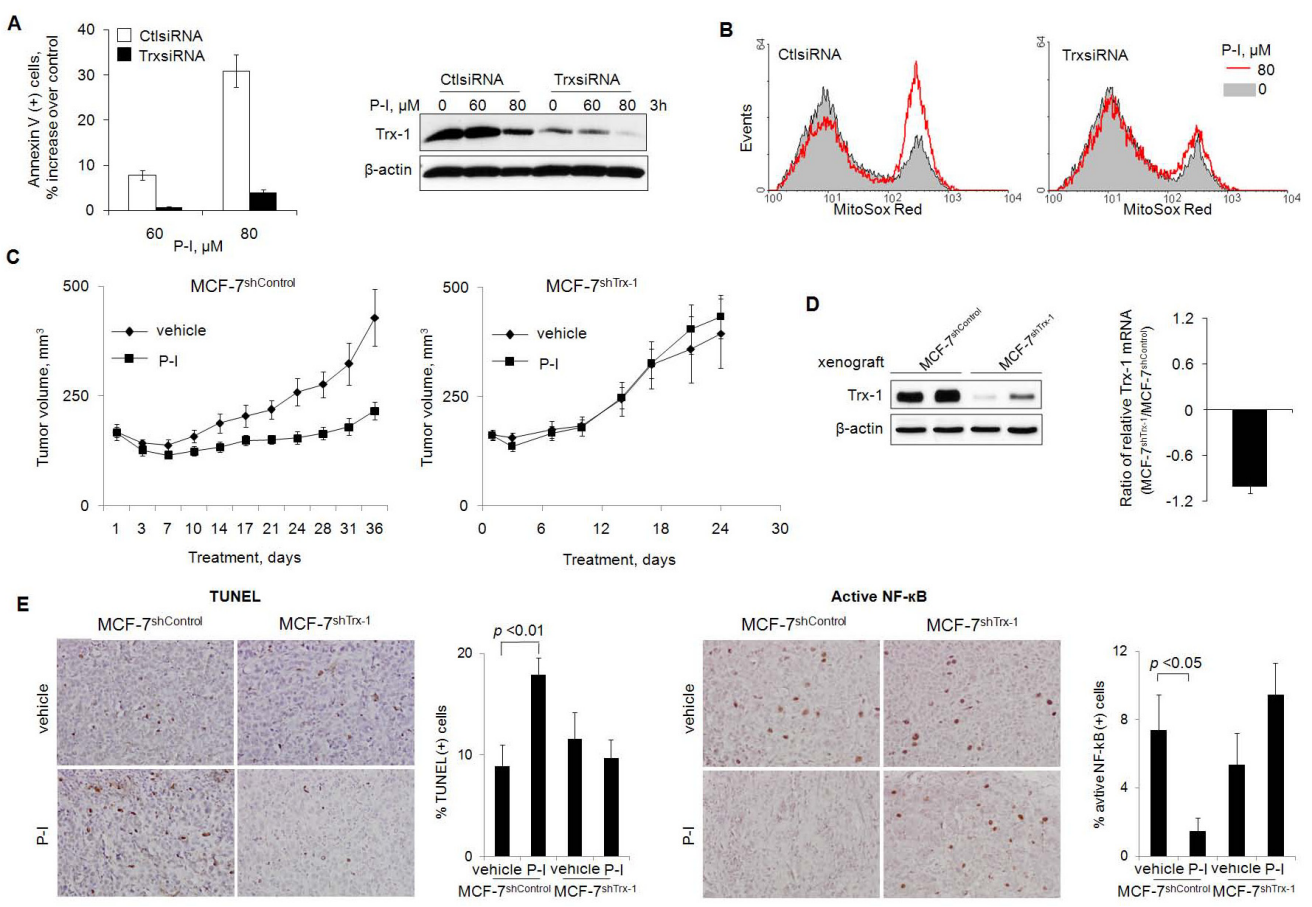
**Thioredoxin-1 mediates the anti-cancer effect of phospho-ibuprofen *in vitro *and *in vivo***. **(A) **MCF-7 cells transfected with thioredoxin-1 (Trx-1) siRNA (TrxsiRNA) or control siRNA (CtlsiRNA) for 72 hours were treated with phospho-ibuprofen (P-I) for 16 hours. Cell death was assayed after annexin V staining. Immunoblots on the right shows the Trx-1 level in these cells. **(B) **Transient transfected MCF-7 cells were assayed for mitochondrial superoxide anion (O_2_^-^) after treatment with P-I for 1 hour. **(C) **Permanent Trx-1 knockdown (MCF-7^shTrx-1^) and its control (MCF-7^shControl^) cells were xenografted into nude mice, which were treated with P-I or vehicle. Tumor growth curves are shown. **(D) **At the end point, Trx-1 expression in vehicle-treated tumors was determined by immunoblot for protein level or by quantitative PCR for mRNA level. **(E) **Cell death (TUNEL) and active NF-κB xenografts as above were determined by immunohistochemistry. Representative images (200×) from each group and quantification results are shown as indicated.

To further assess the role of Trx-1 in the anti-breast-cancer effect of P-I, we silenced the expression of Trx-1 in breast cancer cells using specific siRNA and determined whether P-I-induced cell death was affected. Compared with control cells, the induction of apoptosis by P-I 60 μM or 80 μM was reduced by 91% and 87%, respectively, in Trx-1-depleted cells (Figure 6A). Similar results were obtained with MDA-MB231 cells (Additional file 4). As expected [46], there were parallel changes in the levels of O_2_^-^. P-I failed to induce mitochondrial O_2_^- ^in Trx-1 knockdown cells (Figure 6B).

*In vivo*, we xenografted nude mice with MCF-7^shTrx-1 ^cells (Trx-1 stably knocked down) or with MCF-7^shControl ^cells (control shRNA) (see Materials and methods and Additional file 5). When the average tumor volume was 162 mm^3 ^and 168 mm^3^, respectively, animals were treated with P-I 400 mg/kg/day or vehicle. P-I failed to inhibit the growth of MCF-7^shTrx-1 ^xenografts during the 24 days of observation. In contrast, compared with the vehicle-treated group, P-I inhibited the growth of MCF-7^shControl ^xenografts by 126% (*P *< 0.05; Figure 6C). The nearly complete suppression of Trx-1 levels in these tumors was confirmed by immunoblotting and quantitative PCR (Figure 6D). The effect of P-I on tumor growth reflects its differential cytokinetic effect. Compared with vehicle-treated groups, P-I failed to alter the rates of apoptosis or proliferation in MCF-7^shTrx-1 ^xenografts, whereas in MCF-7^shControl ^xenografts P-I increased apoptosis by twofold (*P *< 0.01) and suppressed proliferation by 49% (*P *< 0.05; Figure 6E and 6 data not shown).

In these tumors, NF-κB activation showed a similar response to P-I: compared with the vehicle-treated group, there was no statistically significant change in MCF-7^shTrx-1 ^xenografts but there was an 80% reduction in xenografts expressing Trx-1 normally (*P *< 0.03; Figure 6E). The inactivation of NF-κB by P-I requires the presence of Trx-1. These results strongly support the notion that the anti-breast-cancer effect of P-I is tightly modulated by Trx-1, the key molecule to turn on the redox signaling.

## Discussion

Our results demonstrate that the novel compound P-I is a strong agent against breast cancer, especially when formulated in liposomes, and establish the critical role of the Trx system in mediating this effect through downstream redox signaling.

The effect of P-I against breast cancer is: broad, encompassing both ER-positive and triple-negative human xenografts; strong, as evidenced by the tumor stasis and tumor regression achieved in MDA-MB231 and MCF-7 xenografts, respectively; and potentially clinically relevant, as these two cell lines represent the most frequent and the most difficult-to-treat subtypes of breast cancer. Our efficacy study also demonstrated that, in agreement with current understanding [31], formulating P-I in liposomes enhanced its anti-cancer efficacy.

The anti-cancer effect of P-I clearly reflects its cytokinetic effect, which consists of inhibition of proliferation and induction of apoptosis, both noted *in vitro *and *in vivo*. In keeping with the differential anti-tumor efficacy, Lipo-P-I had a more pronounced cytokinetic effect than P-I.

Oxidative stress emerges as a key mechanism of action for several anti-cancer agents, and we have observed this with compounds structurally related to P-I [47,48]. Indeed, oxidative stress is a very early change that culminates in cell death via apoptosis and necrosis [49]. In the two breast cancer cell lines we studied, P-I induced oxidative stress and its inhibition by NAC blocked the effect on cell death.

The Trx system plays a pivotal role in redox homeostasis. P-I had a profound effect on the two protein members of this system, Trx-1 and TrxR. In cultured breast cancer cells, P-I drove Trx-1 towards its oxidized form and reduced the expression of Trx-1 in xenografts. P-I also suppressed the activity of TrxR, determined by an assay using purified enzyme and also in cultured and xenografted breast cancer cells treated with P-I. In contrast to Trx-1, the expression of TrxR was not affected by P-I. Of interest, in MCF-7 xenografts Lipo-P-I (which was more efficacious in tumor growth inhibition than free P-I) suppressed Trx-1 expression and TrxR activity to a greater extent than free P-I, suggesting that this effect may be part of the mechanism of action for P-I. These findings substantiate a major suppressive effect of P-I on the Trx system through effects on Trx-1 and TrxR.

The effect of P-I on the Trx system had repercussions on signaling cascades dependent on it, as exemplified by its effects on NF-κB and MAPK. First, the activity of NF-κB was suppressed by P-I in both cultured cells and tumor xenografts. NF-κB is particularly responsive to changes in the Trx system through its Cys62 of p50, whose oxidation renders NF-κB incapable of binding to DNA [12]. The importance of the inactivation of NF-κB following treatment with P-I is indicated by the suppressed expression in xenografts of Bcl-2 and Mcl-1, two anti-apoptotic proteins transcriptionally regulated by NF-κB [44,45]. The MAPK pathway, also regulated by P-I, is downstream of Trx-1. ASK1 is normally bound to Trx-1, keeping the pathway inactive - but when Trx-1 is oxidized, ASK1 is released from the complex, activatingthe downstream MAPK cascade [14]. Of the three main branches of MAPKs, JNK was uniformly activated by P-I in both cell lines and their xenografts. The response of the other two varied in a cell-type-dependent manner. P-I clearly has a context-dependent effect on MAPKs.

**Figure 7:**
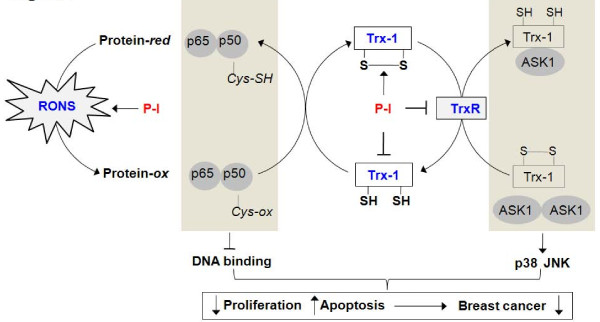
**Effect of phospho-ibuprofen against breast cancer: proposed mechanism of action**. In this model, phospho-ibuprofen (P-I) suppresses breast cancer growth through its dual effect on: reactive oxygen and nitrogen species (RONS), inducing oxidative stress; and the thioredoxin (Trx) system, inhibiting the thioredoxin reductase (TrxR) activity, oxidizing thioredoxin-1 (Trx-1) and suppressing its expression. These effects lead to decreased cell proliferation and increased apoptosis, their net result suppressed breast cancer growth or even tumor regression. Specifically, P-I-induced RONS convert proteins from their reduced (Protein-red) to oxidized (Protein-ox) state. Trx-1, Trx-1-(SH)_2_, reduces its oxidized client proteins - itself, however, being oxidized in the process (Trx-1-S_2_). TrxR recycles Trx-1-S_2 _back to its normal reduced state. The shaded areas explain how the effect of P-I on redox signaling (NF-κB and apoptosis signal-regulating kinase 1 (ASK1)-p38/JNK) is controlled by the central Trx system.

While the modulating effect of P-I on these two signaling mechanisms that are downstream of the Trx system is clear, its direct linkage to the Trx system required the study of xenografts with permanently suppressed Trx-1. P-I suppress the activation of NF-κB only when the expression of Trx-1 was intact. Furthermore, its ability to induce apoptosis and suppress proliferation in these xenografts, two effects heavily mediated by NF-κB and MAPK, was abrogated when the expression of Trx-1 was markedly knocked-down by shRNA. Collectively, these data establish the Trx system as an important mediator of the anti-cancer effect of P-I. Figure 7 outlines the role of P-I in these interactions.

Our findings indicate the role of the Trx system in ensuring cell survival in the face of oxidative stress. As amply demonstrated here, agents such as P-I that paralyze this vital control system enhance cell death and inhibit cell proliferation. The final result of any intervention directed at the Trx system probably depends on complex interactions beyond the NF-κB and MAPK cascades. Nevertheless, in agreement with work by others [4,20,50], this system represents an important target for the development of anti-cancer agents.

## Conclusions

The present work establishes P-I as an agent with a strong effect against breast cancer in a preclinical model, suggests that the mode of its delivery may be important for its efficacy, and establishes the Trx system as a critical mediator of its mechanism of action. The Trx system, vital for cell homeostasis, emerges as an important target for the development of novel anti-cancer agents.

## Abbreviations

ASK1: apoptosis signal-regulating kinase 1; Bcl-2: B-cell lymphoma 2; COX-2: cyclooxygenase-2; DCFDA: 2',7'-dichlorofluorescein diacetate; ER: estrogen receptor; ERK: extracellular signal-regulated kinase; GFP: green fluorescent protein; HER2: human epidermal growth factor receptor-2; HPLC: high-performance liquid chromatography; IC_50_: concentration that inhibits cell growth by 50%; JNK: Jun N-terminal kinase; Lipo-P-I: liposome-encapsulated phospho-ibuprofen; MAPK: mitogen-activated protein kinase; Mcl-1: myeloid cell leukemia 1; MTT: methylthiazolyldiphenyl-tetrazolium bromide; NAC: *N*-acetyl-cysteine; NF: nuclear factor; NSAID: nonsteroidal anti-inflammatory drug; O_2_^-^: superoxide anion; PAGE: polyacrylamide gel electrophoresis; P-I: phospho-ibuprofen; PCR: polymerase chain reaction; RONS: reactive oxygen and nitrogen species; shRNA: short hairpin RNA; siRNA: small interfering RNA; Trx: thioredoxin; Trx-1: thioredoxin-1; TrxR: thioredoxin reductase; TUNEL: terminal deoxynucleotidyl transferase-mediated dUTP nick end labeling.

## Competing interests

LMR and KV declare that they have no competing interests. DK has received compensation from Medicon Pharmaceuticals, Inc. BR has an equity position in Medicon Pharmaceuticals, Inc. BR, LH, GGM and YS have filed patent applications related to the study drug. The authors have no nonfinancial competing interests.

## Authors' contributions

YS conceived the study, participated in its design, carried out most of the *in vitro *and *in vivo *studies, analyzed the data and participated in the preparation of the manuscript. LMR performed the western blots and part of the immunohistochemistry assays for *in vivo *studies. LH carried out the *in vitro *COX-2 expression experiments. GGM carried out the NF-κB electrophoretic mobility shift assays. KV synthesized batches of P-I. DK participated in study design and data analysis, provided P-I, and reviewed the manuscript. BR conceived the study, participated in its design, supervised the work, analyzed data and participated in writing the manuscript. All authors read and approved the final manuscript.

## Supplementary Material

Additional file 1 **Cell kinetic effect of P-I in MDA-MB231 cells**. Cell death (A) and proliferation (B) were examined by Annexin V/PI staining or BrdU staining, respectively, after MDA-MB231 cells were treated with or without P-I for 16 hours. Click here for file

Additional file 2 **P-I affect redox status and COX-2 in breast cancer cells**. (A) MDA-MB231 cells treated with P-I for 1 hour were stained with DCFDA and their fluorescent intensity was determined by flow cytometry. (B) Glutathione (GSH) content of MDA-MB231 cells treated with P-I for 3 hours was determined in cell lysates (*P < 0.01). (C) COX-2 was determined by immunoblot in MCF-7 cells treated with P-I. Click here for file

Additional file 3 **P-I oxidized Trx-1 in MDA-MB231 cells**. The redox status of Trx-1 in MDA-MB231 cells treated with P-I for 1 hour was determined as in Materials and methods. ox, oxidized form; red, reduced form. Click here for file

Additional file 4 **Trx-1 modulates P-I-induced cell death in MDA-MB231 cells**. MDA-MB231 cells were transfected with Trx-1 or control siRNA for 72 hours and then treated with P-I for 16 hours. Cell death was evaluated by annexin V staining. P-I-induced cell death is shown as the percentage of annexin V(+) cells over control (*P < 0.01). Click here for file

Additional file 5 **GFP level in stable Trx-1 knockdown MCF-7 cells**. MCF-7 cells were stably transfected with three Trx shRNA (Trx-C1, Trx-C2 and Trx-C3) or control shRNA in SMART vectors containing GFP. The transfection efficiency was determined by comparing the levels of Trx-1 protein (western blot; upper panel) or endogenous GFP (flow cytometry; lower panel) in stable cell lines with the wild-type (WT) cell line. Click here for file
