# Supplementary figures and images for: Phospho-ibuprofen (MDC-917) suppresses breast cancer growth: an effect controlled by the thioredoxin system
Source: Breast Cancer Res. 2012 Jan 31;14(1):R20. doi: 10.1186/bcr3105 (PMC3496138; doi:10.1186/bcr3105)

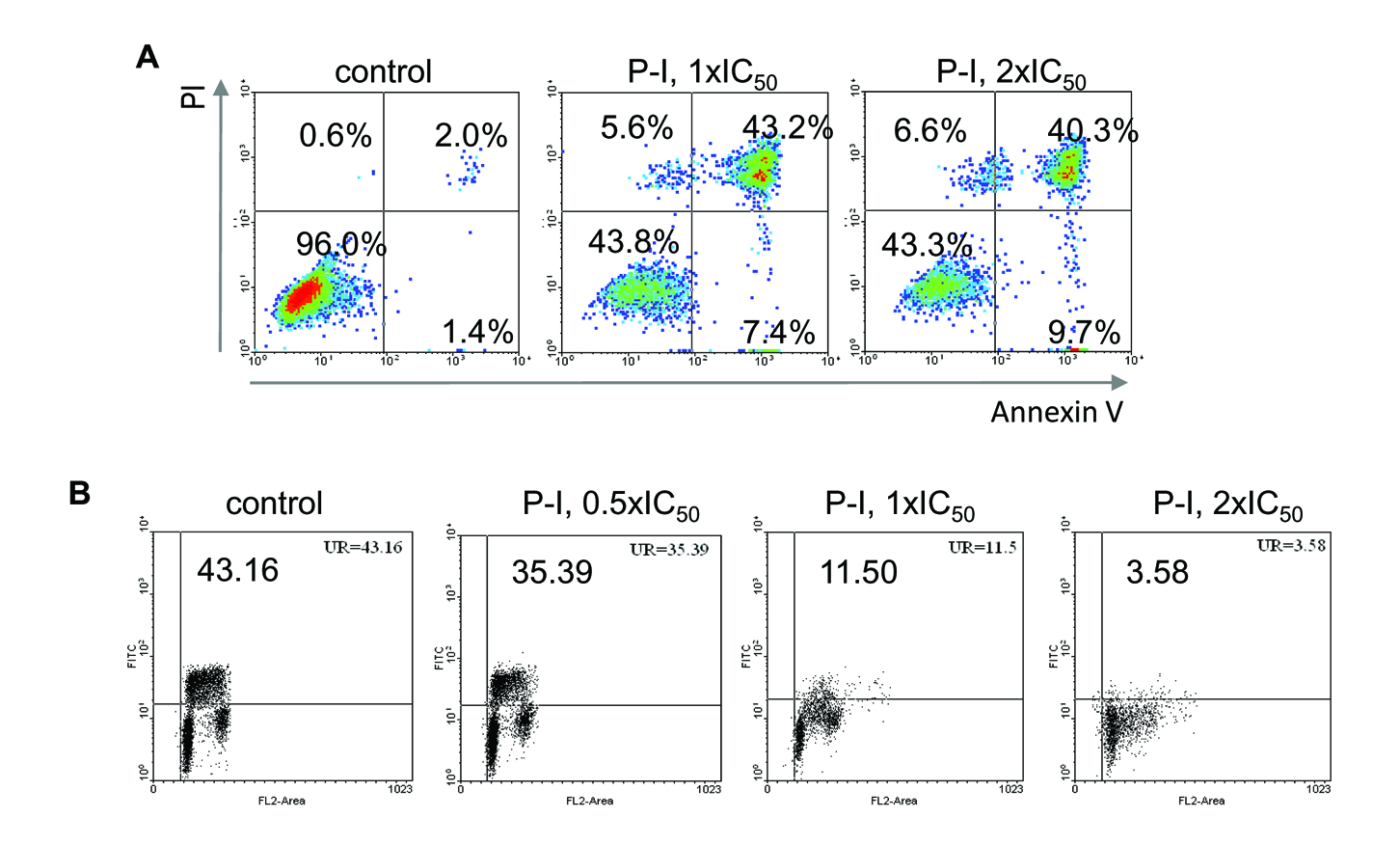

Supplement: Additional file 1 — Cell kinetic effect of P-I in MDA-MB231 cells. Cell death (A) and proliferation (B) were examined by Annexin V/PI staining or BrdU staining, respectively, after MDA-MB231 cells were treated with or without P-I for 16 hours. [file bcr3105-S1.TIFF]

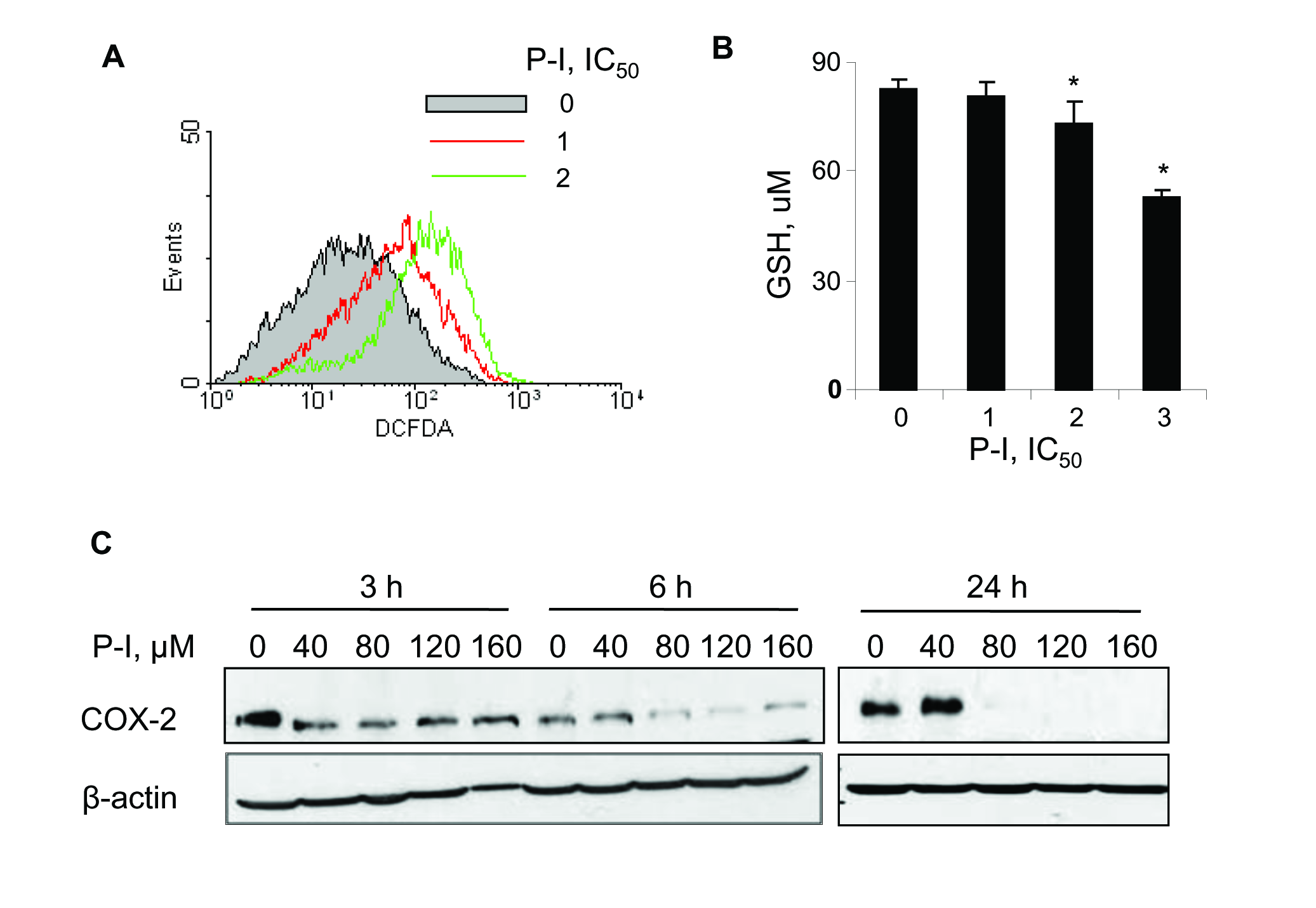

Supplement: Additional file 2 — P-I affect redox status and COX-2 in breast cancer cells. (A) MDA-MB231 cells treated with P-I for 1 hour were stained with DCFDA and their fluorescent intensity was determined by flow cytometry. (B) Glutathione (GSH) content of MDA-MB231 cells treated with P-I for 3 hours was determined in cell lysates (*P < 0.01). (C) COX-2 was determined by immunoblot in MCF-7 cells treated with P-I. [file bcr3105-S2.TIFF]

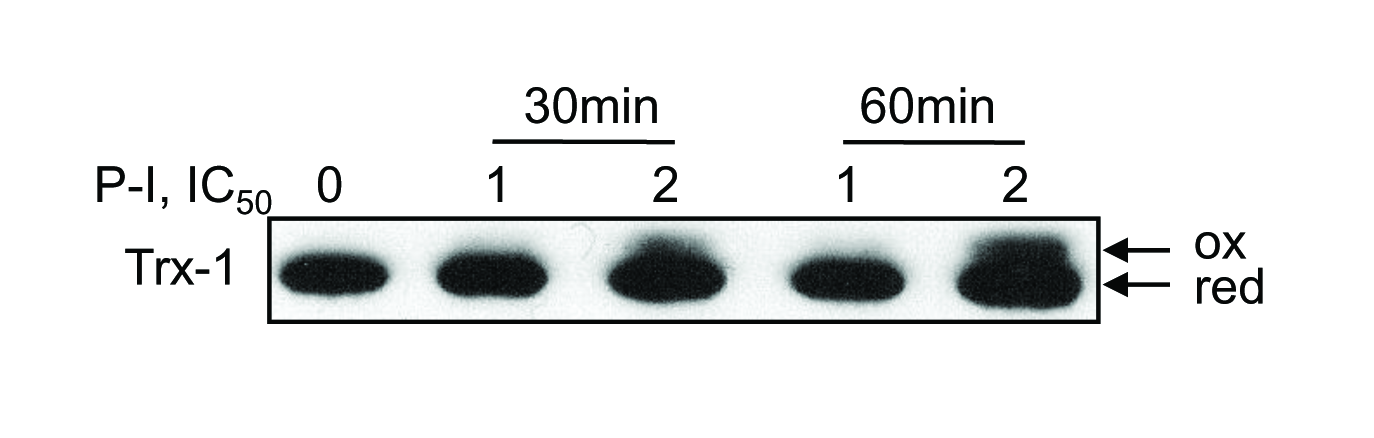

Supplement: Additional file 3 — P-I oxidized Trx-1 in MDA-MB231 cells. The redox status of Trx-1 in MDA-MB231 cells treated with P-I for 1 hour was determined as in Materials and methods. ox, oxidized form; red, reduced form. [file bcr3105-S3.TIFF]

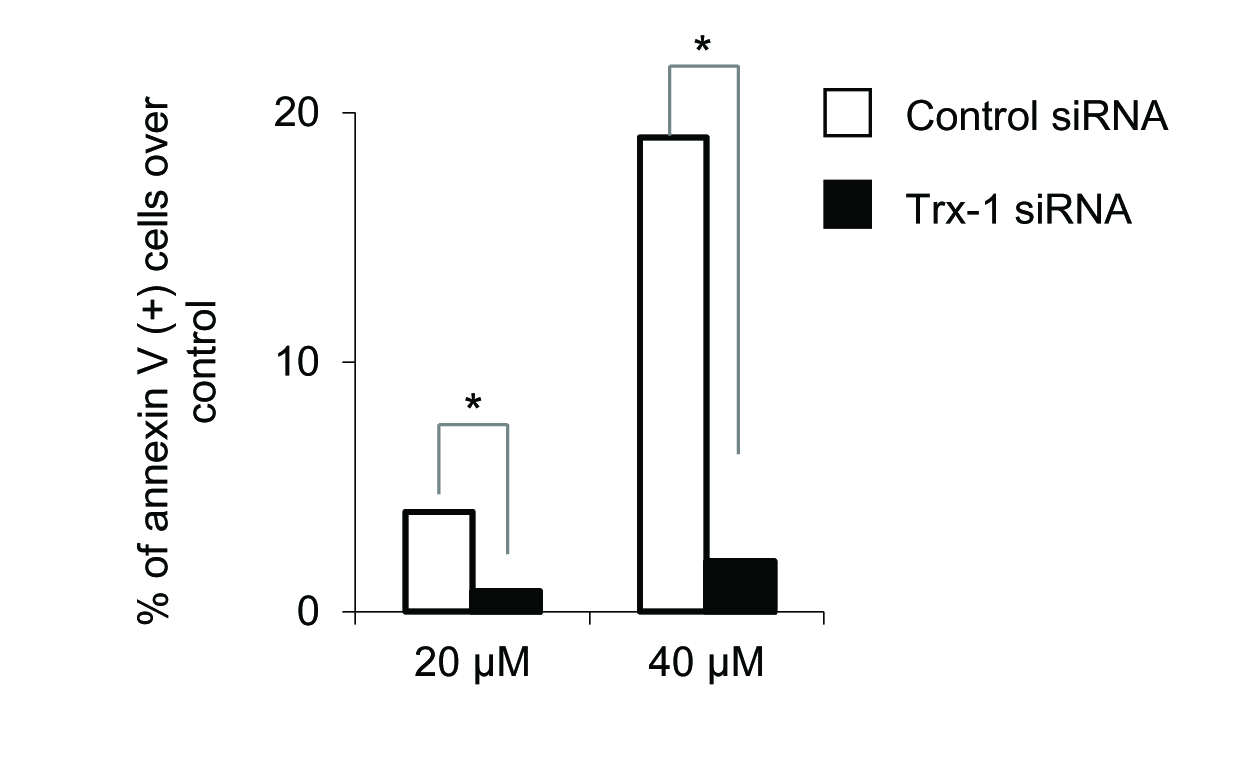

Supplement: Additional file 4 — Trx-1 modulates P-I-induced cell death in MDA-MB231 cells. MDA-MB231 cells were transfected with Trx-1 or control siRNA for 72 hours and then treated with P-I for 16 hours. Cell death was evaluated by annexin V staining. P-I-induced cell death is shown as the percentage of annexin V(+) cells over control (*P < 0.01). [file bcr3105-S4.TIFF]

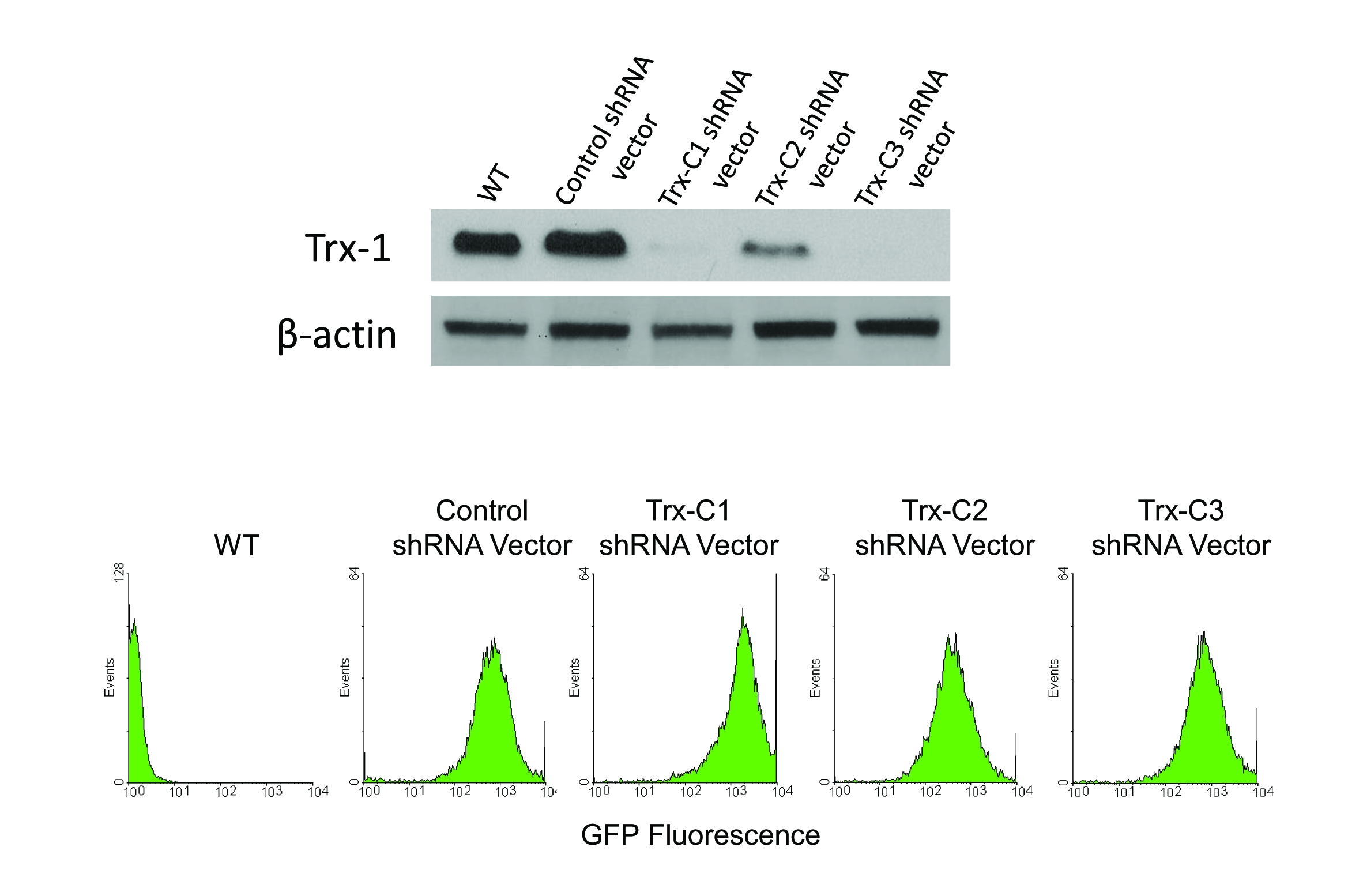

Supplement: Additional file 5 — GFP level in stable Trx-1 knockdown MCF-7 cells. MCF-7 cells were stably transfected with three Trx shRNA (Trx-C1, Trx-C2 and Trx-C3) or control shRNA in SMART vectors containing GFP. The transfection efficiency was determined by comparing the levels of Trx-1 protein (western blot; upper panel) or endogenous GFP (flow cytometry; lower panel) in stable cell lines with the wild-type (WT) cell line. [file bcr3105-S5.TIFF]
